# The Role of Mitochondria-Associated Endoplasmic Reticulum Membranes in Fibrotic Diseases

**DOI:** 10.14336/AD.2025.1087

**Published:** 2026-10-14

**Authors:** Fei Wang, Han Wang, Yun chao Zhang, Cao Dan, Yi Ming Qin, Mei Juan Xie, Xiao Yan Lyu

**Affiliations:** ^1^Department of Dermatology, West China Hospital, Sichuan University, Chengdu, China.; ^2^Laboratory of Dermatology, Clinical Institute of Inflammation and Immunology, Frontiers Science Center for Disease-Related Molecular Network, West China Hospital, Sichuan University, Chengdu, China

**Keywords:** Fibrotic, diseases, Mitochondria-associated, endoplasmic, reticulum, membranes, inflammation, cell, death, cellular, senescence

## Abstract

Fibrotic diseases are characterized by high incidence and mortality rates, posing substantial challenges to global public health due to their considerable disease burden. Mitochondrial damage is a common feature in all fibrotic diseases. Mitochondria do not function in isolation; rather, they are often influenced by stress signals from adjacent organelles. Mitochondria and the endoplasmic reticulum are frequently studied as a subfunctional unit. Mitochondria-associated endoplasmic reticulum membranes (MAM) serve as physical connectors between mitochondria and the endoplasmic reticulum. MAM plays a pivotal role in multiple pathological and physiological processes, including lipid metabolism, inflammation, mitochondrial function, cell death, and cellular senescence. These pathological processes are also implicated in the progression of fibrotic diseases. In this review, we examine the relationship between MAM and key pathological mechanisms in fibrotic diseases, such as cell death, cellular senescence, and inflammation. We further explore the potential of MAM as diagnostic biomarkers and therapeutic targets for fibrotic diseases, thereby offering novel research directions and treatment strategies aimed at improving outcomes for patients with fibrotic diseases.

## 1. Introduction

Pathological fibrosis can occur in all organs, leading to fibrotic diseases or fibroproliferative disorders [1]. Fibrotic diseases encompass a range of conditions including hepatic fibrosis, pulmonary fibrosis, renal fibrosis, peritoneal fibrosis, and dermal fibrosis. The significant prevalence and associated mortality of fibrotic diseases impose a considerable economic burden. The annual combined incidence of major fibrosis-related diseases is approximately 4,968 cases per 100,000 person-years [2]. In developed nations, fibrotic diseases contribute to approximately 45% of disease-related mortality. In contrast, developing countries likely experience even higher incidence and mortality rates for these conditions [3]. These clinical data underscore the urgency of elucidating the molecular mechanisms underlying fibrosis. Although each fibrotic disease presents with organ-specific pathological manifestations, they collectively share common features, most notably the deposition of extracellular matrix (ECM) and excessive collagen secretion. Despite efforts to understand the mechanisms and development of fibrotic diseases, the pathogenesis remains incompletely elucidated. Recent advances highlight organelle dysfunction as a central pathological hallmark of fibrotic diseases.

Among these dysfunctional organelles, mitochondria emerge as a central player due to their dual roles in energy metabolism and cell fate regulation [4]. Dysregulation of mitochondrial homeostasis is a prevalent characteristic observed across a diverse array of fibrotic diseases [5-9]. Therefore, elucidating the mechanisms underlying the disruption of mitochondrial homeostasis is critically important for enhancing our understanding of the pathogenesis of fibrotic disorders and for the development of novel therapeutic strategies. Mitochondria do not exist in isolation; they frequently interact with surrounding organelles and are often studied as a functional subunit alongside the endoplasmic reticulum. The mitochondria-associated endoplasmic reticulum membrane (MAM) acts as both the structural foundation and functional tether linking mitochondria with the endoplasmic reticulum. This interaction enables the stress status of one organelle to influence the other, thereby collectively regulating cellular homeostasis [10]. The MAM is a dynamic structure formed by direct contact between the mitochondrial outer membrane and the ER. Its structural features can be quantified by parameters such as contact length and inter-membrane spacing [11]. The unique architecture of the MAM serves as a crucial platform for intracellular metabolite exchange and signal transduction. It plays a central regulatory role in lipid metabolism, Inflammation, cell death, cellular calcium homeostasis, and redox balance [12, 13]. Recent studies have provided additional evidence for the functions of MAM, such as regulating mitochondrial function, pyroptosis, ferroptosis, and cellular senescence. Dysfunction mediated by MAM directly affects fibroblast biological behavior, particularly redox imbalance and cellular senescence. The transition of fibroblasts to myofibroblasts is a critical pathogenic mechanism in fibrosis, directly driving aberrant collagen synthesis and excessive ECM production [14]. Fibroblast activation is often influenced by inflammation and oxidative stress, with inflammatory cells secreting cytokines that induce this activation [15]. The extracellular oxidative stress microenvironment facilitates the activation and proliferation of interstitial fibroblasts. The depletion of glutathione peroxidase-3 (GPX3) leads to increased production of reactive oxygen species (ROS), thereby augmenting this microenvironment and contributing to the promotion of renal fibrosis [16]. The importance of cell death in fibrotic diseases has been extensively studied [17-19]. In addition to oxidative stress and cell death, cellular senescence represents another pivotal mechanism driving fibrosis progression. Senescent cells secrete the senescence-associated secretory phenotype (SASP), which includes IL-1 and IL-6. These cytokines can transform fibroblasts into myofibroblasts and promote ECM deposition, thereby accelerating fibrosis [20].

This study focuses on the functions of MAM. Given the critical roles of inflammation, cellular senescence, and cell death in the pathological processes of fibrotic diseases, this research explores the potential regulatory effects of MAM on inflammation, cellular senescence, and cell death in fibrotic diseases. Additionally, it explores the therapeutic and diagnostic potential of targeting MAM-associated proteins in fibrotic diseases.

## 2. The structural composition and function of MAM

Mitochondria and the ER are highly dynamic organelles. The MAM is formed by membrane segments from the mitochondrial outer membrane and the ER [11]. The distance between mitochondria and the ER typically ranges from 10 to 60 nm. While classical studies suggested MAMs form when mitochondria-ER proximity reaches <30 nm [21], recent evidence reveals functional MAM assembly even at 10-50 nm distances [22]. This discrepancy may relate to technical variations in measurement methodologies. More than four decades ago, transmission electron microscopy investigations of hepatocyte mitochondria initially suggested the presence of physical connections between the ER and mitochondria [21, 23]. Subsequently, two decades later, an electron tomography study elucidated the existence of diverse tethers with varying morphologies and dimensions that connect the outer mitochondrial membrane (OMM) to specialized subdomains of the ER [24]. MAM is closely associated with inflammation, lipid metabolism, mitochondrial function, programmed cell death and cellular senescence (Fig. 1A).

### 2.1 MAM and lipid metabolism

Interorganellar communication is crucial for lipid metabolism, as different organelles handle various lipid processes [25, 26]. The MAM region, rich in sphingolipid synthases like serine palmitoyltransferase (SPT) and ceramide synthase (CerS), is key for sphingolipid synthesis [27]. Multiple proteins at MAMs regulate lipid metabolism. For instance, UBXD8 depletion, which increases ER-mitochondria contact, raises the proportion of mono- or di-unsaturated phosphatidylcholine (PC) acyl tails and elevates levels of diacylglycerols (DGs), triacylglycerols (TAGs), and lysophospholipids (LPLs). In Huh7 cells, altering Grp75/HSPA9 and MFN2 expression impacts MAM integrity. leading to ER stress and subsequent effects on triglyceride and cholesterol accumulation through divergent pathways [28]. Notably, MAMs not only regulate lipid metabolism but are also modulated by lipid dynamics. Recent studies show that the p97 AAA-ATPase and its adaptor protein UBXD8 control MAMs. Depletion of the p97-UBXD8 complex results in increased lipid saturation and reduced mitochondria-ER contact sites, indicating that these sites are highly sensitive to membrane lipid composition [29]. A high-fat diet can alter MAM structure, leading to prolonged calcium channel opening and mitochondrial calcium overload [30] (Fig. 1B and Table 1). However, our understanding of the system-wide regulatory role of lipids in ER-mitochondria communication remains incomplete. Key unanswered questions include: which type of lipid is more sensitive to the formation of MAM, and how do lipids and cellular states (such as hypoxia, division, senescence, and apoptosis) collectively influence mitochondrial-ER membrane contact sites^+^

**Figure 1.**
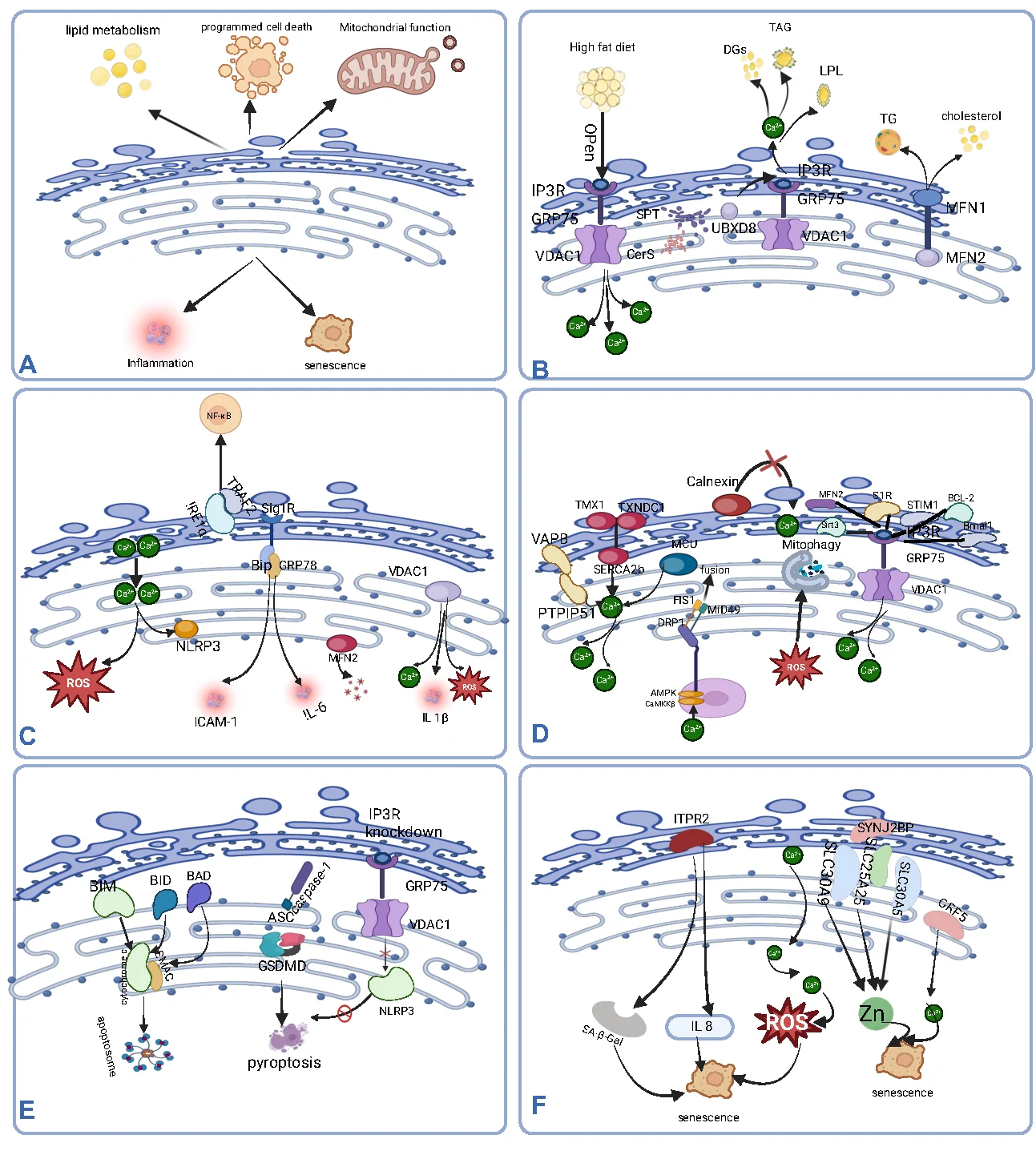
**The function of MAM**. (**A**) The overall function of MAM. (**B**) MAM and lipid metabolism. The figure shows that multiple ion channel complexes are involved in lipid metabolism, such as IP3R-GRP75-VDAC1 and MFN1-MFN2. (**C**) MAM and Inflammation, the figure shows that inflammatory pathways involve multiple proteins, such as IRE1α, GRP78, Sig1R and VDAC1. (**D**) MAM and Mitochondrial function. There are two main calcium channel complexes, VABP-PTPIP51 and IP3R-GRP75-VDAC1. Multiple proteins such as Sirt3, MFN2, STIM1, and Bcl2 can regulate the mitochondrial function of IP3R-GRP75-VDAC1. ROS modulates mitophagy. (**E**) MAM and programmed cell death. BIM, BID, and BAD are involved in apoptosis. Reducing IP3R1, which lowers calcium transfer from the endoplasmic reticulum to mitochondria, decreases inflammasome activation and inhibits pyroptosis. (**F**) MAM and Cellular senescence. MAM protein ITPR2 and the transport of calcium and zinc ions on MAM are involved in cellular senescence.

### 2.2 MAM and inflammation

MAM is vital for inflammasome formation and pathogen recruitment, playing a key role in inflammatory responses [31]. The MAM facilitates Ca²^+^ transfer from the ER to mitochondria, which induces mitochondrial calcium overload, ROS generation, and inflammasome activation [32]. However, the critical threshold at which Ca²^+^ flux transitions from physiological signaling to pathological stress remains unelucidated. Additionally, MAM affects NF-κB transcriptional activity. During ER stress, the unfolded protein response triggers interactions involving IRE1α and TRAF2, forming the IRE1α-TRAF2 complex. This complex is crucial for phosphorylating and degrading IkBα, enabling NF-kB to enter the nucleus and enhance pro-inflammatory cytokine transcription [33]. Proteins on MAM, such as BiP/GRP78, have a significant impact on inflammation. The inhibition of sigma-1 receptor (Sig1R) can modulate BiP/GRP78 activity, which exacerbates sepsis-induced lung injury by reducing the expression of ICAM-1 and IL-6, as well as neutrophil infiltration [34]. Furthermore, voltage-dependent anion channel (VDAC) proteins on the MAM regulate inflammation, VDAC1 aiding mitochondrial Ca²^+^ uptake and ROS production [35, 36]. VDAC1 knockdown suppresses IL-1β inflammasome assembly [37]. Mitofusin 2 (MFN2), a key protein on the mitochondrial outer membrane at the MAM, is essential for its structure [38]. Activating MFN2 helps prevent MAM disruption and reduces liver inflammation [39] (Fig. 1C and Table 1). However, some studies contradict this view, suggesting that the distance between the ER and mitochondria increases upon MFN2 knockout [40]. The underlying causes of these contradictory findings remain unresolved. Future studies may need to employ diverse detection methods across different cell types to better characterize these changes. Novel MAM proteins involved in inflammatory regulation are currently being characterized, with many more awaiting discovery.

### 2.3 MAM and Mitochondrial function

MAM can regulate mitochondrial function by influencing mitochondrial metabolism, dynamics, mitophagy, and calcium fluxes. Studies indicate that MAM, a specialized ER subdomain, is vital for oxidation in mitochondria [41, 42]. Structural and functional integrity of the MAM is crucial for glucose metabolism and pyruvate use in mitochondrial functions [43]. Glycolytic enzymes such as hexokinase 2 (HK2) are enriched within MAM [44]. In ALS models, defective pyruvate metabolism is linked to poor MAM formation^[41]^. MAM is crucial for mitochondrial metabolism and dynamics. PRRSV infection in pigs raises mitochondrial calcium levels, activating the CaMKKβ-AMPK-DRP1 signaling pathway, which interacts with FIS1 and MiD49 to enhance mitochondrial fusion [45]. Mitophagy, a selective autophagic process, serves as a quality control mechanism by removing damaged or superfluous mitochondria, thereby maintaining mitochondrial network integrity [46]. Mitochondria face constant exposure to ROS and various stressors, potentially causing dysfunction. Damaged mitochondria are selectively removed through mitophagy, mitophagy is critical for preventing dysfunction [47]. MAM is a vital center for lipid regulation and also plays a significant role in ion metabolism. Calcium ions, primarily stored in the ER, are essential for activating the mitochondrial permeability transition pore (mPTP) [48]. The ER has been consistently recognized as the primary reservoir of calcium ions [49]. The MAM harbors proteins that both promote and suppress Ca²^+^ transfer from the ER to mitochondria. Calnexin regulates the balance of cytosolic and MAM-associated calcium by reducing its availability for mitochondrial uptake and enhancing its retention in the ER [50]. TMX1/TXNDC1 interacts with SERCA2b, reducing ER Ca²^+^ uptake while facilitating Ca²^+^ flux at the MAM [51]. The mitochondrial calcium uniporter (MCU), along with MICU1 and MICU2, governs Ca²^+^ transfer between the ER and mitochondria [52, 53]. Ca²^+^ release from the ER through the inositol 1,4,5-trisphosphate receptor (IP3R) channels target the VDAC on the outer mitochondrial membrane (OMM). Once across the OMM, Ca²^+^ enters the mitochondrial matrix through the MCU on the inner membrane [54]. Additionally, the cytosolic chaperone Grp75 strengthens the IP3R-VDAC interaction, thereby enhancing mitochondrial Ca²^+^ uptake [55]. The IP3R-GRP75-VDAC complex is regulated by proteins such as MFN2, S1R, and STIM1. MFN2 activation promotes ER-mitochondria proximity, increasing mitochondrial calcium levels [56]. The Sigma-1 receptor (S1R), a chaperone protein at the MAM, stabilizes IP3R to facilitate calcium transfer from the ER to mitochondria, thereby regulating mitochondrial calcium levels [57]. In Sig-1R knockout mice, the distance between the ER and mitochondria widens [58]. Stromal interaction molecule proteins 1 and 2 (STIM1 and STIM2), located on the ER membrane, are crucial for regulating intracellular calcium signaling and maintaining calcium homeostasis [59]. The store-operated calcium entry (SOCE) channel serves as the primary route for for Ca²^+^ influx in non-excitable cells [60]. STIM1 can activate SOCE, modulating calcium flux through the IP3R1-GRP75-VDAC1 complex (for details, see the beginning of this section) [61]. The BCL-2 family proteins, key apoptosis regulators, are divided into three main categories based on their structure and function [62]. This family includes anti-apoptotic proteins like Bcl-2 and Bcl-xL, and pro-apoptotic proteins like Bax and Bak [63]. In doxorubicin-induced cardiac injury, Bcl-2 activation reduces calcium overload via the IP3R-VDAC1-MCU pathway [64]. Bmal1 and SIRT3 also regulate calcium signaling, with Bmal1 reducing mitochondrial calcium overload and MAM formation [65], and SIRT3 alleviating mitochondrial calcium overload and diabetes-related cognitive decline [66]. Beyond the IP3R1-GRP75-VDAC1 complex, other novel calcium channel complexes are also being discovered. Studies show that MCU deletion in cardiac ischemia-reperfusion (I/R) mice can limit mitochondrial Ca²^+^ overload and prevent irreversible opening of the mPTP [67]. However, some researchers have demonstrated that the opening of the mPTP shows no significant difference after MCU deletion in mouse hearts [68]. Recent studies show that the VAPB-PTPIP51 complex regulates calcium ion channels [69]. VAPs (type II transmembrane proteins) mediate ER-mitochondria communication and include VAPA and VAPB [70]. PTPIP51, a mitochondrial protein, binds VAPB to enhance Ca²^+^ transfer from the ER to mitochondria via the IP3R-GRP75-VDAC1 complex [71, 72], [73]. Among MAM-related proteins, three of four contain an uncharacterized domain termed the synaptotagmin-like mitochondrial lipid-binding (SMP) domain, SMP domains conserved from yeast to humans [74]. PDZD8, an SMP-domain protein, influences Ca²^+^ dynamics; its overexpression boosts mitochondrial Ca²^+^ uptake in dendrites, while knockdown reduces it (Fig. 1D and Table 1) [75]. Sphingosine-1-phosphate (S1P) is now recognized as pivotal for MAM calcium signaling [76]. In degenerated intervertebral discs, S1P levels drop sharply, its inhibition or knockdown accelerates nucleus pulposus cell degeneration [77]. A deficiency in S1P causes protein accumulation in the ER, leading to its expansion, increased ER-mitochondria contact, disrupted calcium transfer, and mitochondrial dysfunction [78]. In summary, MAM plays a crucial role in regulating mitochondrial function, but research gaps exist. For instance, exploring whether the interaction between MAM and other organelles (such as lysosomes and peroxisomes) affects mitochondrial function could alter our understanding of cellular metabolism.

### 2.4 MAM and programmed cell death

MAM plays a role in programmed cell death, primarily through apoptosis and pyroptosis, but also ferroptosis and autophagy. This process is regulated by specific molecular pathways. Apoptosis is divided into extrinsic and intrinsic types. Extrinsic apoptosis is initiated by death receptors on the plasma membrane, such as Fas (CD95), TNFR1, TRAIL-R1 (DR4), and TRAIL-R2 (DR5), which are activated by specific ligands^[79, 80]^. In contrast, intrinsic apoptosis is initiated by intracellular changes. The Bcl-2 protein family is crucial in regulating apoptosis, with pro-apoptotic members (BIM, BID, BAD, NOXA, PUMA) and anti-apoptotic members (Bcl-2, Bcl-xL, Mcl-1, Bcl-W) [63]. Experimental evidence suggests that the activity of certain pro-apoptotic members (e.g., BID) may depend on post-translational modifications (such as phosphorylation), a regulatory layer that has not been adequately considered in most existing models [81]. Pro-apoptotic proteins trigger mitochondrial outer membrane permeabilization (MOMP), releasing cytochrome c and forming the apoptosome with SMAC, which activates caspases to start apoptosis [82]. Mitochondrial outer membrane permeabilization (MOMP) has traditionally been considered the 'point of no return' in the process of cell death. Nevertheless, recent findings have demonstrated that necroptosis can proceed independently of mitochondrial permeability transition [83], thereby challenging this long-established paradigm. Proteins localized at MAM, such as P53, PML, and PTEN, are also involved in the regulation of this process [84-86]. The activated inflammasome sensor ASC, in collaboration with caspase-1, assembles into a functional inflammasome complex. Caspase-1 plays a crucial role in the proteolytic cleavage of the pore-forming protein gasdermin D (GSDMD) [87]. The transfer of calcium ions between the mitochondria and the ER is implicated in the activation of the NLRP3 inflammasome. Empirical evidence indicates that the knockdown of IP3R1 disrupts calcium ion transport (refer to Section 2.3 for further details), resulting in diminished inflammasome activation and a subsequent reduction in pyroptosis (see Fig. 1E and Table 1) [88].

### 2.5 MAM and Cellular senescence

The ER and mitochondria are integral to the regulation of calcium ions. Current research suggests that disturbances in calcium ion balance within the ER can trigger molecules associated with the senescence-associated secretory phenotype [89]. Proper cytoplasmic calcium regulation is essential for cell cycle control, and imbalances in mitochondrial calcium can result in cell cycle arrest [90]. In the context of oncogene-induced senescence, a reduction in ITPR2 levels has been shown to decrease senescence markers, such as SA-β-Gal activity and interleukin 8 expression [91]. Notably, during senescence, the expression levels of ITPR1, ITPR2, and ITPR3 remain relatively unchanged, indicating that their activity, rather than expression, is of greater importance [92]. Furthermore, the stable expression of ITPR family proteins during cellular senescence suggests that current research may have overlooked critical post-translational modifications, thereby exposing significant gaps in our understanding. Elevated calcium ion levels in mitochondria can decrease mitochondrial membrane potential and boost ROS production, ultimately contributing to cellular senescence [93]. Zinc ions also regulate mitochondrial membrane potential and function, influencing cellular senescence [94, 95]. MAM plays a critical role in regulating both calcium and zinc ion homeostasis. Zinc transport proteins, such as SLC30A9, SLC25A25, and SLC30A5 aiding proton transfer from the ER to mitochondria [96]. In intervertebral disc degeneration, the overexpression of SYNJ2BP has been shown to mitigate the aging of nucleus pulposus cells by improving mitochondria-ER contact and zinc ion levels [97]. Lipid metabolism at the MAM is implicated in cellular aging processes. ORP5, an ER membrane protein involved in phospholipid exchange, promotes cellular senescence by increasing mitochondrial calcium uptake when its expression is downregulated (Fig. 1F and Table 1) [98].

**Table 1:** Mitochondria-associated endoplasmic reticulum membrane-related genes and their functions.

Role	Protein	Functions
**lipid metabolism**	UBXD8	increases endoplasmic reticulum-mitochondria contact sites, results in cellular lipidomes characterized by elevated levels of mono- or di-unsaturated phosphatidylcholine (PC) acyl tails
	Grp75 MFN2	influences triglyceride (TG) and cholesterol accumulation
**Inflammation**	IRE1α	allowing NF-κB to translocate into the nucleus
	Sig1R	downregulation of intercellular adhesion molecule-1 (ICAM-1), interleukin-6 (IL-6) levels, and neutrophil infiltration
	VDAC1	reduces the formation of the IL-1β inflammasome
	Mfn2	mitigates the disruption of MAM, consequently alleviating hepatic inflammation
**Mitochondrial function**	HK2	regulating glucose metabolism
	CaMKKβ DRP1 FIS1	Mitochondrial fission and fusion
	TMX1/TXNDC1	reducing ER Ca²^+^ uptake and enhancing
	SERCA2b	Ca²^+^ flux at the MAM
	MCU	modulates Ca²^+^ transfer between the ER and mitochondria
	MFN2 S1R STIM1	enabling calcium ions to flow from the endoplasmic reticulum to mitochondria
	BCL-2 Bmal1 SIRT3	through the IP3R-VDAC1-MCU signaling pathway
	S1P	mitochondrial dysfunction
**programmed cell death**	Bcl-2 family	apoptosis
	P53, PML, and PTEN	apoptosis
	IP3R1	pyroptosis
**Cellular senescence**	ITPR2	associated with senescence: senescence-associated beta-galactosidase (SA-β-Gal) activity and interleukin 8 expression
	SYNJ2BP	mitochondrial zinc ion homeostasis
	ORP5	promotes cellular senescence by enhancing mitochondrial calcium uptake

Bcl-2:B-cell lymphoma 2; Bmal1:Brain and muscle ARNT-like 1; CaMKKβ:Calcium/calmodulin-dependent protein kinase kinase β; DRP1:Dynamin-related protein 1 (DNM1L); FIS1:Fission 1 (mitochondrial outer membrane protein); Grp75:Glucose-regulated protein 75 (HSPA9, mortalin); HK2:Hexokinase 2; IRE1α:Inositol-requiring enzyme 1α (ERN1); IP3R1:Inositol 1,4,5-trisphosphate receptor type 1; ITPR2:Inositol 1,4,5-trisphosphate receptor type 2; MCU:Mitochondrial calcium uniporter; MFN2:Mitofusin 2; ORP5:Oxysterol-binding protein-related protein 5 (OSBPL5); P53:Tumor protein p53 (TP53); PML:Promyelocytic leukemia protein; PTEN:Phosphatase and tensin homolog; S1P:Sphingosine-1-phosphate; S1R:Sigma-1 receptor (Sig1R); SERCA2b:Sarco/endoplasmic reticulum Ca²^+^-ATPase 2b; SIRT3:Sirtuin 3; STIM1:Stromal interaction molecule 1; SYNJ2BP:Synaptojanin-2-binding protein; TMX1/TXNDC1:Thioredoxin-related transmembrane protein 1; UBXD8:UBX domain-containing protein 8 (UBXN8); VDAC1:Voltage-dependent anion channel 1.

## 3. The role of MAM in fibrotic diseases

Fibrotic diseases are characterized by intricate and multifaceted mechanisms. Central to the pathogenesis of various fibrotic conditions are processes such as inflammation, cellular senescence, and cell death, with MAM playing a crucial role in these pathophysiological mechanisms. This section aims to elucidate the relationship between MAM and fibrosis within these pathological contexts.

### 3.1 The role of MAM in inflammation associated with fibrotic diseases

Fibrotic diseases, including chronic kidney disease, liver cirrhosis, idiopathic pulmonary fibrosis, cardiac fibrosis, and cutaneous fibrosis, are characterized by persistent inflammation [99-102]. Current research underscores the pivotal role of inflammation in the pathogenesis of these conditions. For instance, pulmonary fibrosis frequently originates from acute lung injury, during which inflammation enhances capillary permeability and causes alveolar damage [103, 104]. Fibrosis is associated with inflammasome formation and inflammatory factors, with inflammasomes facilitating the production of these mediators. Alterations in MAM can influence inflammasome assembly. In liver disease, innate immune cells activate inflammasomes through Toll-like receptors (TLRs) and the nuclear factor kappa-light-chain-enhancer of activated B cells (NF-κB) pathway, leading to the release of cytokines such as interleukin-1β and tumor necrosis factor-α, which exacerbate fibrosis and may result in organ failure [105]. In chronic kidney disease, intestinal inflammation and barrier weakening permit the translocation of bacterial toxins, thereby initiating inflammation and worsening renal fibrosis [106]. Skin and immune cells possess receptors that trigger inflammation following injury, potentially exacerbating scarring [107]. Prolonged inflammation worsens scarring, but depleting macrophages in LysMCre/iDTR mice reduces it [108]. Both normal and excessive scarring may involve inflammasomes [109, 110], which are complexes within immune cells that detect danger signals and activate caspase-1. This activation leads to the production of proinflammatory cytokines IL-1β and IL-18, thereby exacerbating inflammation [111]. While fibrosis in the liver, kidney, and lungs is reliant on the activation of the NLRP3 inflammasome, the pathways through which this activation contributes to fibrosis in various fibrotic diseases exhibit unique characteristics. Upon activation, NLRP3 facilitates the release of several inflammatory cytokines. In the context of liver and pulmonary fibrosis, this process is primarily linked to the activation of IL-1β [105, 112]. Furthermore, IL-18, a downstream effector of the NLRP3 inflammasome, has been shown in murine models of left ventricular pressure and volume overload to promote myocardial fibrosis and ventricular remodeling [113]. In renal fibrosis, the activation of the NLRP3 inflammasome in podocytes is crucial for driving fibrosis in diabetic kidney injury [114]. While fibrotic mechanisms vary by organ, they commonly involve inflammasome activation, leading to continuous cytokine release and excessive ECM deposition. Macrophages significantly contribute to ECM secretion and remodeling by producing matrix metalloproteinases (MMPs) and tissue inhibitors of metalloproteinases (TIMPs) [115]. MAM influence fibrosis by regulating the inflammasome, controlling inflammatory macrophages, and orchestrating ECM remodeling. Damage to MAM disrupts the VDAC1/GRP75/IP3R complex, causing a Ca²^+^ imbalance (see Section 2.3 for details). This imbalance affects inflammatory mediator permeability and NLRP3 inflammasome production [116]. Thus, targeting MAM and inflammasomes could be a therapeutic approach for fibrotic diseases. DRP1, localized at the mitochondria-associated endoplasmic reticulum membrane (MAM), plays a crucial role in regulating prostaglandin E2 (PGE_2_) production in inflammatory macrophages. PGE_2_ subsequently modulates fibroblast activation, thereby influencing the progression of fibrosis [29]. In addition, MAM proteins related to ECM remodeling include MMP-2. MMP-2 functions as an effector molecule within the inflammatory microenvironment, and its activity is regulated by the inflammatory environment. MMP-2 is a key enzyme involved in the degradation of collagen and other ECM components. It has been shown that MMP-2 localizes to MAM and cleaves calreticulin in a concentration-dependent manner. Calreticulin can limit mitochondrial calcium overload, thereby reducing mitochondrial damage [117]. These findings suggest that modulation of MAM may influence ECM remodeling by regulating MMP-2 2 activity, which subsequently affects mitochondrial calcium flux. Nonetheless, within the domain of fibrotic diseases, there remains a paucity of systematic research concerning the precise molecular mechanisms through which MMP2, localized to mitochondria-associated membranes (MAMs), contributes to the progression of fibrosis via the regulation of mitochondrial function. In-depth exploration of this regulatory pathway will help elucidate novel mechanisms underlying fibrogenesis (Fig. 2).

### 3.2 The role of MAM in programmed cell death associated with fibrotic diseases

Programmed cell death represents a form of cell death regulated by a multitude of proteins [118]. Common types of programmed cell death include apoptosis, pyroptosis, and ferroptosis [119]. These mechanisms play a pivotal role in the onset and progression of fibrotic diseases. The occurrence of cell death can lead to cellular damage, which may subsequently provoke inflammatory responses, activate fibroblasts, and stimulate the secretion of substantial amounts of ECM components, such as collagen, ultimately culminating in fibrosis [120, 121]. Phosphofurin acidic cluster sorting protein-2 (PACS2), located on the MAM, is crucial for maintaining mitochondrial integrity and MAM structure; its deficiency causes mitochondrial fragmentation [122]. Type II alveolar epithelial cells are crucial in the context of pulmonary fibrosis. In individuals with idiopathic pulmonary fibrosis, these cells demonstrate mitochondrial and ER stress, along with reduced PACS2 expression. Augmenting PACS2 expression ameliorates mitochondrial-ER interactions and reduces apoptosis in pulmonary fibrosis [123]. Hepatic fibrosis arises when liver injury activates hepatic stellate cells (HSCs) to produce ECM and collagen [124]. Reversing fibrosis may involve the death of activated HSCs. Different programmed cell death affects liver fibrosis progression and regression, depending on the cell type. Specifically, autophagy in HSCs promotes fibrosis, while autophagy in liver macrophages reduces it [125]. TMEM41B, a protein on the MAM, is crucial; its deficiency leads to liver fibrosis by reducing mitochondria-ER contact, while increasing TMEM41B can fix hepatic autophagy issues [126]. BAG3 binds to BCL-2 family proteins like BCL-2 and BAX to inhibit mitochondrial pathway apoptosis, acting as an anti-apoptotic gene. Elevated BAG3 in systemic sclerosis patients suggests a link to skin fibrosis [127].

**Figure 2.**
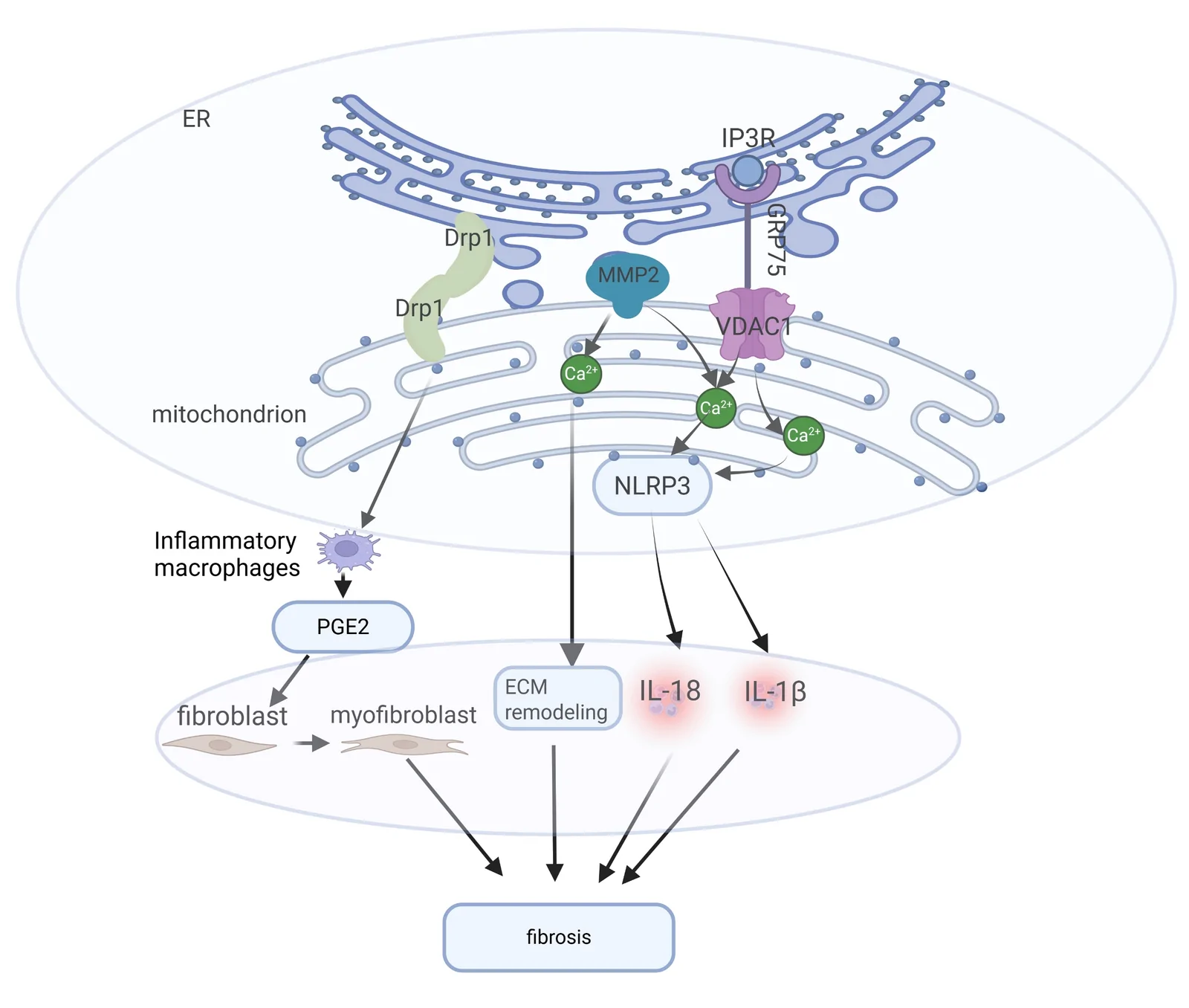
**The role of MAM in inflammation associated with fibrotic diseases**. DRP1 regulates prostaglandin E2 (PGE_2_), PGE_2_ subsequently modulates fibroblast activation, thereby influencing the progression of fibrosis. MMP-2 regulates the degradation of collagen and other ECM, thereby influencing the progression of fibrosis. The calcium channel complex influences fibrosis by modulating the release of inflammatory mediators.

Research has demonstrated that Bcl2 affects MAMs by modulating IP3R activity [65]. BAG3's interaction with Bcl2 implies a potential role in MAM and skin fibrosis, warranting further research. However, in cardiac fibrosis, BAG3 expression is reduced, leading to decreased autophagy and promoting fibrosis [128], challenging the view of BAG3 as solely anti-fibrotic. It prompts us to reevaluate the temporal dynamics of BAG3 regulatory networks, suggesting it may exert opposing roles in early-stage fibrosis (anti-apoptotic) versus late-stage fibrosis (pro-inflammatory). Pyroptosis, influenced by inflammasomes like NLRP3, is crucial in fibrosis [129, 130], with NLRP3 being the most extensively studied inflammasome. The NLRP3 inflammasome is capable of recognizing danger signals and subsequently activating pro-inflammatory caspase-1, which processes the precursors of IL-1β, IL-18, and Gasdermin D (GSDMD) into their mature forms [131]. The expression of p66Shc is elevated in liver fibrosis, and its inhibition is associated with the alleviation of fibrosis [132]. In liver fibrosis, p66Shc overexpression activates NLRP3, but its role in pyroptosis and fibrosis remains unexplored [133]. Exploring their potential relationship could enhance understanding of liver fibrosis mechanisms. In some scleroderma patients, skin lesions exhibit GSDMD protein expression, whereas prolonged inflammation show minimal change. A similar increase occurs in a bleomycin-induced scleroderma mouse model, where GSDMD knockout reduces skin fibrosis [134]. Pyroptosis is intricately linked with MAM. In cardiomyocytes, ER stress increases MAM presence, disrupting calcium transport and causing mitochondrial damage. This damage raises mitochondrial ROS levels, activating the NLRP3 inflammasome and inducing pyroptosis [135].

**Figure 3.**
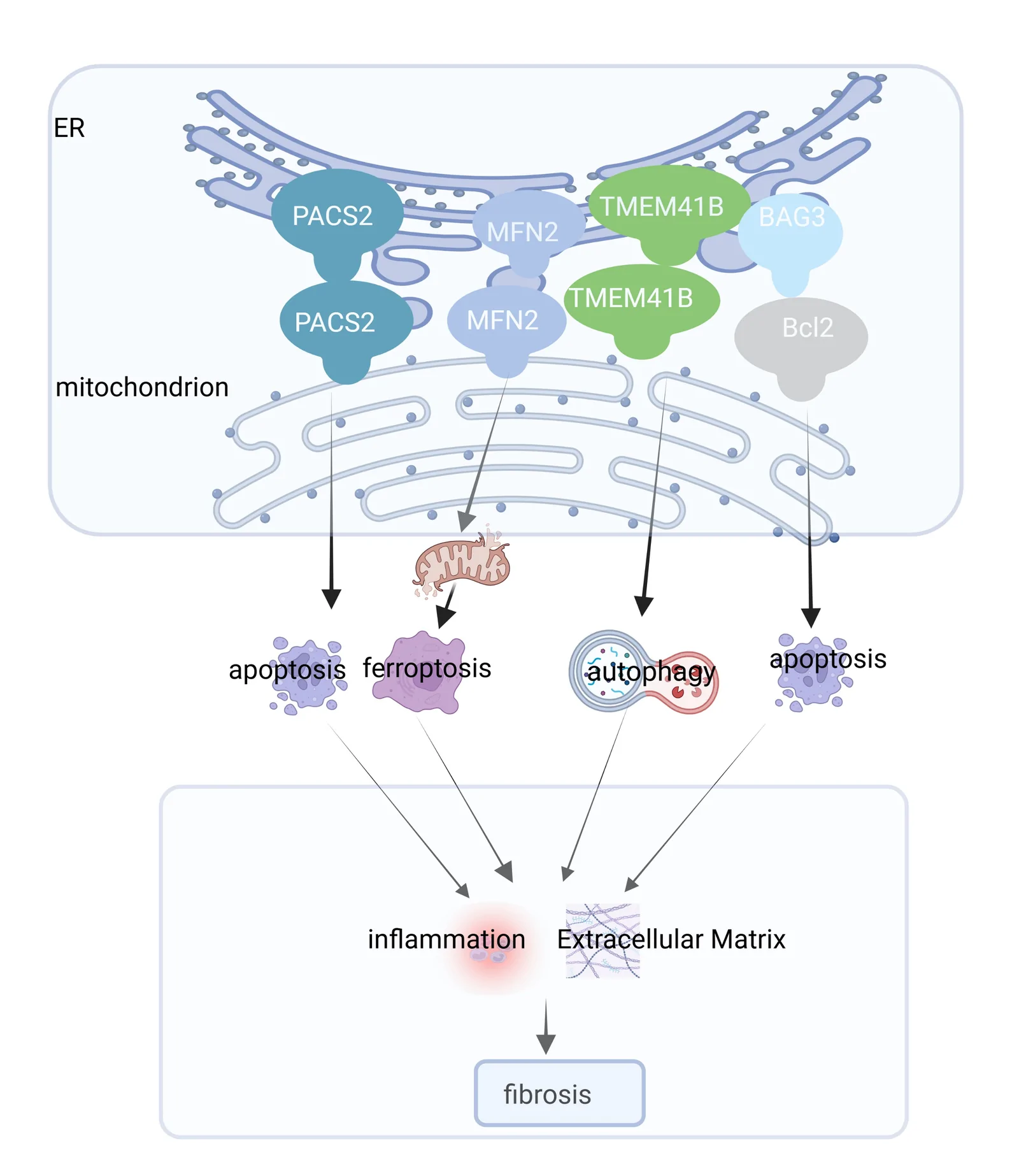
**The Role of MAM in programmed cell death associated with fibrotic Diseases**. PACS2, MFN2, TMEM41B, and BAG3 play pivotal roles in cellular processes, with PACS2 regulating apoptosis, MFN2 modulating ferroptosis, TMEM41B governing autophagy, and BAG3 controlling apoptosis. Together, these proteins orchestrate inflammation and the remodeling of the extracellular matrix, thereby contributing to the regulation of fibrosis.

Homocysteine triggers ER stress, boosts ER-mitochondria communication, and disrupts calcium balance, activating the NLRP3 inflammasome and causing pyroptosis. Using calcium chelator BAPTA and calcium channel blockers reduces macrophage pyroptosis [136], highlighting MAM's role in this process. Targeting MAM may reduce fibrosis by influencing pyroptosis. The link between MAM and pyroptosis in fibrotic diseases is a new area with no existing studies. Fibrosis and ferroptosis share metabolic pathways, with glutaminolysis in activated fibroblasts promoting ferroptosis via the TCA cycle [137]. Disrupted iron and redox balance significantly affect TGF-β-induced epithelial-mesenchymal transition (EMT) in fibrosis by increasing intracellular free iron and ROS, enhancing TGF-β's pro-fibrotic effects [138]. The sigma-1 receptor on MAMs can be targeted by CGI1746 to inhibit ferroptosis [139]. PERK, a MAM-associated protein, regulates ferroptosis by linking ER stress to ER-mitochondria communication by interacting with outer mitochondrial membrane proteins like MFN2. Inhibiting the PERK/ATF4/CHAC1 pathway in diabetic nephropathy strengthens ER-mitochondria connections, reducing ferroptosis [140]. Consequently, MAM is crucial for ferroptosis, particularly during alveolar epithelial cell injury in pulmonary fibrosis. Mitofusin-2 (Mfn-2), a GTPase involved in the formation of ER-mitochondria contacts, regulates MAMs and participates in ferroptosis during alveolar epithelial cell injury [141]. However, in fibrotic diseases, the relationship between MAM and ferroptosis in liver fibrosis and skin fibrosis remains unexplored, suggesting a potential area for future research (Fig. 3 and Table 2).

**Table 2:** The role of MAM associated proteins in programmed cell death associated with fibrotic diseases.

Fibrosis Type	Key MAM Targets	Programmed Cell Death Pathways	Established Mechanisms
**Pulmonary**	PACS2 Mfn-2	Apoptosis Ferroptosis	PACS2 deficiency disrupts mitochondrial-ER crosstalk; Mfn-2 regulates ferroptosis in AECs
**Hepatic**	TMEM41B	Autophagy	TMEM41B loss impairs MAM integrity
**Cardiac**	BAG3	autophagy	BAG3 downregulation reduces autophagy, promoting cardiac fibrosis
**Dermal**	GSDMD	Pyroptosis	The GSDMD pores induce calcium ion (Ca²^+^) leakage in the MAM domain, thereby exacerbating NLRP3 inflammasome activation.
**Renal**	PERK	ferroptosis	inhibiting the PERK/ATF4/CHAC1 pathway strengthens MAM, reducing ferroptosis

PACS2: Phosphofurin acidic cluster sorting protein 2; MFN2: Mitofusin 2; TMEM41B: Transmembrane Protein 41B. BAG3: Bcl-2-associated athanogene 3. GSDMD: Gasdermin D. PERK: Protein Kinase R-like Endoplasmic Reticulum Kinase.

### 3.3 The role of MAM in cellular senescence related to fibrotic diseases

Aging is a complex biological process intricately associated with the onset of various diseases, particularly fibrotic conditions. Aging leads to significant changes in lung gene expression, notably in genes involved in the regulation of redox balance, TGF-β signaling, and the ECM, which may promote fibrotic disease progression. Age-related modifications in the ECM are pivotal in the development of chronic fibrotic diseases [142]. ECM remodeling alters tissue properties and affects cellular responses to mechanical forces such as compression, tension, and shear stress [143, 144]. As tissues age, mesenchymal cells exhibit growth arrest and resistance to apoptosis, while the ECM undergoes significant compositional and structural changes. These alterations can lead to abnormal cell activation and regulation [145]. Notably, similar senescent fibroblasts appear in both fibrosis and cancer, linking these conditions. These cells can modify the ECM and influence epithelial and immune changes, thereby promoting disease progression [146]. The removal of senescent cells and the ablation of genes associated with senescence have been shown to reverse various forms of fibrosis. Experimental models have shown that the elimination of senescent cells enhances pulmonary function and reverses pulmonary fibrosis [147]. Furthermore, the absence of the p21 gene in mice has been associated with the removal of senescent cells from liver fibrotic scars, thereby ameliorating liver fibrosis [148]. In myocardial ischemia-reperfusion injury, post-injury treatment with the anti-aging drug navitoclax significantly reduces scar size. Proteomic analyses show that the removal of senescent cells mitigates fibrosis and inflammation following injury [149]. As individuals age, the structure and function of MAMs may undergo modifications, potentially impact normal cellular functions and contributing to the onset of age-related diseases [150]. During the aging process, the integrity of MAM may be compromised, leading to a disruption in the signaling pathways between mitochondria and the ER, which adversely affect cellular metabolism and viability [151]. A study employing co-immunoprecipitation has demonstrated that Hst1 promotes diabetic wound healing by regulating the IP3R-GRP75-VDAC1 channel complex to suppress mitochondrial-ER contact [152]. Cardiomyocyte senescence frequently results in cardiac dysfunction, ventricular remodeling, and fibrosis. Studies have shown that quercetin enhances mitochondrial-ER co-localization and reduces the inter-organelle distance, thereby mitigating aging in H9c2 cells [153]. Although these findings reveal potential associations, the majority of conclusions are derived from animal or cell models, necessitating further validation to ascertain their relevance to human pathology. The focus and key cells differ among fibrotic diseases, and current research has only minimally explored the relationship between MAM and cellular senescence. Future investigations should examine this relationship across diverse cell types, including dermal fibroblasts, renal tubular epithelial cells, alveolar epithelial cells, and hepatic stellate cells, to enhance the understanding of fibrotic disease pathogenesis.

### 3.4 The role of MAM in other mechanisms related to fibrotic diseases

Fibroblasts exhibit a high sensitivity to mechanical stimulation. Under pressure overload, fibroblasts have the potential to differentiate into myofibroblasts. Meanwhile, mechanical stress also influences collagen cross-linking and myocardial stiffness [154]. In a dermatological study, a split-face controlled trial employing a novel mechanical stimulation device (Mécano-Stimulation™) revealed that mechanical stimulation of skin and subcutaneous tissue led to increased levels of hyaluronic acid, elastin, type I collagen, and matrix metalloproteinase-9 (MMP-9) in the treated side, along with enhanced fibroblast migration capacity [155]. These findings indicate that mechanical stimulation may modulate skin structure and function by regulating fibroblast activity. Transient Receptor Potential Vanilloid (TRPV) signaling pathway is recognized as a known mechanosensitive pathway. In fibrosis research, the TRPV signaling pathway has been found to play a critical role in fibrotic processes across various tissues. TRPV1 can alleviate renal fibrosis by inhibiting the TGF-β/SMAD signaling pathway [156]. TRPV4 promotes fibroblast-to-myofibroblast transformation in pulmonary and cardiac fibrosis, affecting disease progression [157, 158]. TRPV1, activated by pressure or stimuli, can reduce mitochondrial dysfunction during podocyte injury in diabetic nephropathy by improving mitochondria-ER interactions [159]. Recent studies have also demonstrated that TRPV4 is located at MAM sites, and the presence of TRPV4 can significantly influence basal calcium levels. TRPV4 expression leads to a significant elevation in mitochondrial calcium concentrations [160]. However, beyond modulating mitochondrial calcium through MAMs, whether mechanical signaling via TRPV channels additionally influences lipid alterations to impact fibrotic disease progression remains unexplored in current research. Mechanical signaling pathways regulate the MAM. Fibroblast activation and ECM deposition are closely related to mechanical signals. However, in fibrotic diseases, the differential expression of TRPV subtypes and their specific regulatory mechanisms on the MAM remain understudied.

## 4. Therapeutic targeting of MAMs in fibrotic diseases

Recent investigations into fibrosis and therapies targeting MAM have predominantly focused on renal and pulmonary fibrosis. The pathological interaction between the ER and mitochondria impairs renal tubular cell functionality, resulting in tubular inflammation and fibrosis. In the context of cisplatin-induced acute kidney injury (AKI), cisplatin instigates mitochondrial damage, notably causing the leakage of mitochondrial DNA (mtDNA) into the cytosol. This cytosolic mtDNA activates the ER-resident cyclic GMP-AMP synthase (cGAS)-stimulator of interferon genes (STING) pathway, which subsequently induces tubular inflammation and fibrosis. Notably, inhibition of the STING pathway significantly reduces tubular inflammation and improves outcomes in acute kidney injury [161]. In vivo studies have confirmed that C176 can suppress STING activation, thereby alleviating ER stress and mitochondrial dysfunction, ultimately mitigating renal fibrosis [162, 163]. Additionally, CAE (Citrus aurantium extract) has been shown to reduce the upregulation of protein expression in the PERK pathway, a marker of bleomycin-induced ER stress, thereby mitigating ER stress levels in murine lung tissues. Furthermore, CAE treatment inhibits the activation of ATF3, a downstream protein associated with ER stress induced by bleomycin or TM, while enhancing PINK1 expression levels in type II alveolar epithelial cells (AEC IIs). AEC-IIs are local unipotent stem cells that transform into pulmonary AEC-Is following damage to the cell barrier and help maintain lung tone and nonprofessional phagocytosis in innate immunity and immune balance [164]. The A549 cell line serves as a canonical research model for type II alveolar epithelial cells (AEC II). This intervention effectively rescues TM-induced disruptions in mitochondrial homeostasis within A549 cells [165]. Sigma-1, a protein located in the MAM, plays a significant role in cellular function. Fluvoxamine, a sigma-1 receptor agonist, has been shown to improve pulmonary fibrosis outcomes with efficacy comparable to that of nintedanib [166]. VAPB-PTPIP51 is a newly identified calcium ion channel transport complex on MAM. TUDCA strengthens the VAPB-PTPIP51 interaction, restoring mitochondrial calcium ion levels in lung fibroblasts and easing pulmonary fibrosis [167]. The relationship between MAM-associated proteins and fibrotic diseases is summarized in Table 3. A recent study revealed that patients with liver fibrosis exhibit a significant decrease in MFN2 levels, but tetramethylpyrazine (TMP) can improve hepatic fibrosis by increasing MFN2 and restoring MAM balance [168]. STIM1-KO mice have MAM abnormalities and cardiac fibrosis [169], suggesting STIM1 agonists might treat cardiac fibrosis. There are numerous proteins associated with MAM, and further exploration is needed to uncover more MAM-related proteins linked to fibrosis, which will rely on proteomics and single-cell sequencing technologies. A recent study purified MAM fractions from hypertrophic myocardium and performed quantitative proteomic analysis to identify a new regulatory protein FMO2, highlighting the method's effectiveness in discovering MAM-targeted proteins [170]. Additionally, small-molecule compounds and natural products targeting the MAM proteins are yet to be found. In addition to directly regulating MAMs proteins, advances in nanotechnology have led to therapies targeting mitochondria and the ER to modulate MAMs. Functional modification through charged natural amino acids and peptides is a promising therapeutic approach in mitochondrial-targeted therapy [171]. Recently, researchers developed a mitochondrial-targeted delivery strategy by conjugating a tri-phenylphosphonium cation (TPP+) to the C12 molecule, which significantly enhanced its mitochondrial specificity and bioactivity, which effectively reduced bleomycin-induced pulmonary fibrosis [172]. Due to the weak targeting ability of the ER, current research on ER-targeted drug delivery mainly focuses on ligand modification. Common strategies for ER targeting involve the insertion of peptide sequences such as KDEL, KKXX, RARC, and Eriss [173]. Among these, KDEL is the most extensively studied. As an ER retention signal, it enables recombinant proteins to specifically accumulate in the ER and can be utilized for delivering therapeutic molecules targeting ER-related diseases [174]. Due to the relatively large molecular weight of targeting peptides, nanocarrier-mediated delivery is generally required for precise localization to the ER. Targeted therapy for mitochondria and ER offers potential for minimizing side effects and enhancing therapeutic efficacy. In an in vivo study utilizing KDEL peptide-modified celastrol for ER-targeted therapy of colorectal cancer, the targeted formulation exhibited significantly reduced tumor growth and markedly decreased systemic toxicity compared to its non-targeted counterpart [25].

**Table 3:** Drugs targeting MAM-associated proteins and their relationship with fibrotic diseases.

Drug	MAM-associated targets	Mechanism	Fibrotic diseases	Ref.
**C176**	STING	C176 inhibits STING activation, mitigating renal fibrosis	renal fibrosis	[164,165]
**CAE**	PERK, ATF3	CAE inhibits PERK and ATF3 activation, mitigating Pulmonary fibrosis	Pulmonary fibrosis	[167]
**Fluvoxamine**	sigma-1 receptor	Fluvoxamine improves pulmonary fibrosis via sigma-1 agonis	Pulmonary fibrosis	[168]
**TUDCA**	VAPB-PTPIP51	TUDCA can enhance the binding force between VAPB and PTPIP51	Pulmonary fibrosis	[169]
**TMP**	MFN2	TMP reverses hepatic fibrosis by upregulating MFN2 expression.	liver fibrosis	[170]
**CPS**	PACS2	CPS treatment restores PACS2 levels and alleviates pulmonary fibrosis.	Pulmonary fibrosis	[125]

STING: Stimulator of Interferon Genes; CAE: Citrus aurantium extract; PERK: Protein Kinase R-like Endoplasmic Reticulum Kinase; ATF3: Activating Transcription Factor 3; TUDCA: Tauroursodeoxycholic Acid; VAPB-PTPIP51: Vesicle-associated membrane protein-associated protein B - Protein tyrosine phosphatase interacting protein 51. MFN2: Mitofusin 2; TMP: Tetramethylpyrazine; CPS: capsaicin; PACS2: Phosphofurin acidic cluster sorting protein 2

## 5. Conclusions and prospects

In the majority of fibrotic diseases, oxidative stress is a prevalent characteristic, frequently resulting in ER stress and mitochondrial damage. Mitochondria and ER are increasingly studied as a functional unit, with the MAM serving as a vital structural link between these organelles. This membrane plays a vital role in regulating regulating glucose and lipid metabolism, modulating calcium ion transport, controlling cell death, influencing cellular senescence, and maintaining mitochondrial function.

The complex and highly dynamic nature of the MAM structure present significant challenges for its direct regulation [175]. Currently, the most compelling evidence for MAM formation is derived from observations at the electron microscopy level. In addition to electron microscopy, techniques such as in situ proximity ligation assays and co-immunoprecipitation have been employed to investigate the interactions among proteins that tether the ER to mitochondria [176]. The expression of MAM proteins varies across different fibrotic diseases. With the advancement of scientific technologies, particularly in research methodologies such as proteomics and metabolomics, it is anticipated that more comprehensive insights into the functions of MAM proteins will be achieved. Detailed analyses of the MAM proteome hold the potential to identify additional key proteins linked to fibrotic diseases, as well as their post-translational modifications. This includes examining the effects of S-palmitoylation on protein function and membrane localization within MAM, thereby elucidating the molecular mechanisms underlying MAM involvement in disease pathogenesis [177]. Metabolomics technology is capable of detecting metabolite alterations associated with MAM, clarifying their regulatory roles within cellular metabolic pathways, and identifying novel biomarkers and therapeutic targets for disease diagnosis and treatment [178]. A study employed multiple approaches to enrich mitochondria-ER fractions from human fibroblasts, identifying over 400 compounds involved in membrane composition and specific metabolic pathways with high reproducibility. This advancement enhances the exploration of ER-mitochondria interactions and their significance in metabolic pathways associated with various diseases [96]. Studies have demonstrated that circulating MAM proteins can serve as biomarkers for disease. For example, the expression of the MAM protein GRP78 is significantly increased in individuals with type 2 diabetes and shows a positive correlation with glycated hemoglobin levels. Nevertheless, traditional techniques often inadequately capture alterations in MAM-associated proteins. Conversely, the integration of proteomics and metabolomics facilitates the simultaneous detection of thousands of proteins, providing a more comprehensive representation of pathological states. This integrative approach significantly enhances the identification of disease biomarkers and expedites the development of targeted therapeutics [179]. Future advancements in imaging and ion-tracing technologies could enable real-time observation and enhanced visualization of ion transfer across MAM, providing valuable tools for studying their role in fibrotic diseases. Nonetheless, the regulation of ion channels within the MAM remains debated. Traditionally, Bcl-xL was believed to promote IP3R1 channel opening and support mitochondrial bioenergetics [180]. Nevertheless, The study by Rosa et al. suggests it may inhibit this process [181]. This highlights the complexity of ion channel regulation at MAM and the need for further research into these conflicting findings

Given the highly dynamic nature of MAM and the diversity and organ-specific differences in MAM-related proteins, investigating their role in fibrotic diseases necessitates a multifaceted approach. Live-cell imaging can be utilized to monitor their dynamic changes, proteomics can be employed to identify differential expression of MAM proteins across various fibrotic organs, and parallel single-cell RNA sequencing can be employed to detect cell-type-specific variations in MAM-associated proteins. Nonetheless, the study of the relationship between MAM and fibrosis encounters significant challenges. Current proteomic methodologies typically require protein quantities in the microgram to milligram range, yet the yields of MAM from certain tissue samples are often insufficient to meet these requirements. This necessitates the advancement of more sensitive and miniaturized proteomic technologies to overcome these limitations. The highly dynamic nature and structural complexity of MAM present substantial challenges in the development of precise targeted therapies against their associated proteins. Furthermore, the cell-type specificity and microenvironmental regulation of MAM require highly selective therapeutic strategies to prevent unintended disruption of physiological functions.
